# Relating Global Cognition With Upper-Extremity Motor Skill Retention in Individuals With Mild-to-Moderate Parkinson's Disease

**DOI:** 10.3389/fresc.2021.754118

**Published:** 2021-10-22

**Authors:** Jennapher Lingo VanGilder, Cielita Lopez-Lennon, Serene S. Paul, Leland E. Dibble, Kevin Duff, Sydney Y. Schaefer

**Affiliations:** ^1^School of Biological and Health Systems Engineering, Arizona State University, Tempe, AZ, United States; ^2^Department of Physical Therapy and Athletic Training, University of Utah, Salt Lake City, UT, United States; ^3^Discipline of Physiotherapy, Sydney School of Health Sciences, Faculty of Medicine and Health, The University of Sydney, Sydney, NSW, Australia; ^4^Center for Alzheimer's Care, Imaging and Research, University of Utah Health Sciences Center, Salt Lake City, UT, United States; ^5^Department of Neurology, University of Utah Hospital, Salt Lake City, UT, United States

**Keywords:** motor learning, global cognition, upper-extremity, task-specific training, Parkinson's disease

## Abstract

**Background and Purpose:** Cognition has been linked to rehabilitation outcomes in stroke populations, but this remains unexplored in individuals with Parkinson's disease (PD). The purpose of this secondary data analysis from a recent clinical trial (NCT02600858) was to determine if global cognition was related to skill performance after motor training in individuals with PD.

**Methods:** Twenty-three participants with idiopathic PD completed 3 days of training on an upper-extremity task. For the purposes of the original clinical trial, participants trained either “on” or “off” their dopamine replacement medication. Baseline, training, and 48-h retention data have been previously published. Global cognition was evaluated using the Montreal Cognitive Assessment (MoCA). Linear regression examined whether MoCA score predicted longer-term retention at nine-day follow-up; baseline motor task performance, age, PD severity, depressive symptoms, and group (medication “on”/“off”) were included as covariates. Baseline and follow-up motor task performance were assessed for all participants while “on” their medication.

**Results:** MoCA score was positively related to follow-up motor task performance, such that individuals with better cognition were faster than those with poorer cognition. Baseline task performance, age, PD severity, depressive symptoms, and medication status were unrelated to follow-up performance.

**Discussion and Conclusions:** Results of this secondary analysis align with previous work that suggest cognitive impairment may interfere with motor learning in PD and support the premise that cognitive training prior to or concurrent with motor training may enhance rehabilitative outcomes for individuals with PD. Findings also suggest that assessing cognition in individuals with PD could provide prognostic information about their responsiveness to motor rehabilitation.

## Introduction

Despite clear evidence of deficits in upper extremity motor control and dexterity in Parkinson's disease (PD) (1, 2) that meaningfully impact on one's activities of daily living (3), most rehabilitation research and clinical practice for PD focuses on gait and balance problems. When prescribed, however, motor rehabilitation can improve upper extremity movement patterns and physical function (4, 5), depending on one's ability to learn and retain novel motor skills. While individuals with PD may benefit from physical rehabilitation, they demonstrate slower learning rates (6) and learn to a lesser extent (7) than individuals without PD, yet longer-term skill retention remains unclear (8). In light of this, some people with PD show marked gains following therapeutic intervention, while others do not [e.g. (4), see also (9)]. The ability to predict therapeutic responsiveness could help therapists streamline and personalize treatments. However, most predictive tools or models of post-intervention motor outcomes are time- and cost-intensive [e.g., annual clinical measures (10), neuroimaging (11), etc.].

In contrast, cognitive assessment may be a feasible, brief, and relatively inexpensive tool for gaining insight to an individual's motor learning capacity [see (12)]. Global cognitive status has been shown to predict gains in gait speed following standard-of-care physical therapy independent of primary diagnosis (13, 14). With respect to PD specifically, physical therapy combined with cognitive training may be more efficacious in improving reactive postural adjustments (i.e., responses to perturbations) and motor symptoms than physical therapy alone (15). Furthermore, lower-extremity physical therapy (i.e., aerobic exercises, treadmill training) has been shown to improve global cognition as well in people with PD (16), although combined physical and cognitive therapy may be more beneficial (17). However, the relationship between global cognitive measures and upper-extremity improvements in PD has not been explored. Empirically, visuospatial function has been linked with upper limb motor learning in both younger (18–21) and older adults (22–26) without PD, and since visuospatial deficits can occur with PD (27–29), this may help explain why people with PD tend to learn motor skills slower and to a lesser extent than those without PD. However, the extent to which cognitive impairment (global or specific) interferes with upper extremity motor learning in individuals with PD remains unknown.

In a recent randomized clinical trial in individuals with mild-to-moderate PD (clinicaltrial.gov registration number NCT02600858) (30), motor practice while “on” dopamine replacement medication (i.e., levodopa) improved 48-h retention of a functional upper extremity motor task compared to practice “off” dopamine replacement medication. The purpose of the present study was to perform secondary analyses of these data to evaluate whether cognition was related to skill learning in the upper extremity. Based on previous findings, it was hypothesized that cognitive functioning would be related to longer-term retention of an upper extremity motor task, where better cognition would be associated with more skill retention.

## Methods

### Participants

Twenty-three adults aged ≥50 years old with a confirmed diagnosis of PD were included in this secondary analysis of data from a previously published randomized clinical trial (clinicaltrials.gov registration number NCT02600858) (30). Inclusion criteria included idiopathic PD diagnosed by a neurologist, age 50–80 years, in Hoehn and Yahr stages 1–3, and had been on a stable antiparkinsonian medication regime for 1 month prior to pretest assessment as well as throughout the study. Exclusion criteria included prior surgical treatment of PD (e.g., deep brain stimulation), dementia [Montreal Cognitive Assessment (31) (MoCA) < 18] (32), and the presence of concomitant neurological conditions. Included participants must have been taking dopamine replacement medications. The clinical trial protocol required half the participants (*n* = 12) to complete upper extremity motor training while continuing to take their prescribed dose of levodopa medication; the other half (*n* = 11) skipped their first dose of medication each day of motor training such that they were “off” medication following overnight withdrawal. These participants took their remaining daily doses after they completed the motor training each morning. Details regarding dopamine medication and other participant characteristics have been previously reported (30).

Global cognition was measured using the MoCA, a brief cognitive screening tool in which scores range from zero to 30; a score of 26 (or higher) is considered to be normal cognitive functioning, as defined by the publisher (31). To evaluate upper extremity dexterity, participants completed the Nine-hole peg test (33) (a timed clinical measure of dexterity) and another timed experimental upper extremity dexterity task that simulates buttoning a shirt unimanually (24, 34, 35); for both these tasks faster trial times indicate better performance. Participants were also tested by a trained examiner with the motor subsection of the Movement Disorder Society-Unified Parkinson's Disease Rating Scale (MDS-UPDRS) (total range of scores = 0–152) (36). To evaluate depressive symptoms, participants completed the Geriatric Depression Scale (GDS) Short Form (37), a self-report rating tool consisting of 15 items and a score of four or lower is considered normal. Participants self-reported hand dominance. All participant characteristic data, including MoCA score, were collected in an initial visit while the participants were “on” their prescribed dose of dopamine replacement medication, regardless of which group they were randomized to (“on” vs. “off” medication).

### Upper-Extremity Motor Training

As described previously (30), the motor training protocol required participants to complete a familiarization trial, then 50 training trials each day for three consecutive days. More details regarding the motor task are provided below. Participants were then re-tested 2 and 9 days later. Two-day follow-up was the stated primary outcome of this clinical trial and was therefore reported previously; thus, only the longer-term nine-day follow-up was included in this analysis.

**Figure 1:**
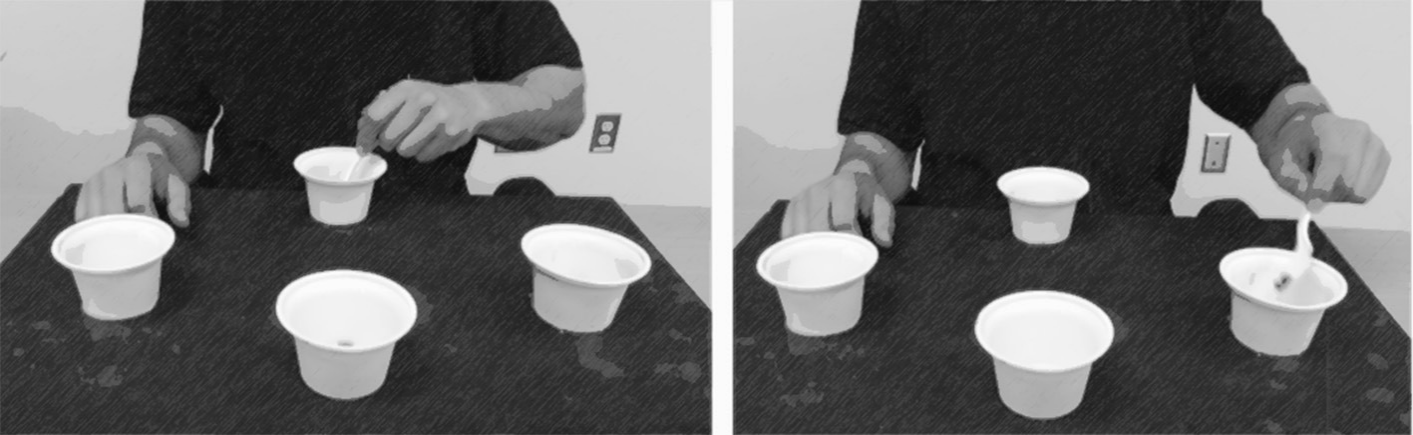
Participants used their non-dominant hand to complete a reaching task that simulates feeding oneself; participants use a spoon to select only two beans from the center “home” cup and deposit them into target cups. One trial consisted of 15 repetitions (i.e., five arcs to each of the three target cups). This figure was adapted from “Dexterity and Reaching Motor Tasks” by MRL Laboratory is licensed under CC BY 2.0.

The motor task used in this study was designed to mimic an activity of daily living [i.e., feeding (38)]. This task has been validated against subjective and objective measures of daily functioning in a cognitively impaired sample (39). The experimental apparatus was comprised of three “target” cups placed 16 cm from a center “home” cup at 45, 90, and 135 degrees (Figure 1). Participants were asked to use a plastic spoon held in their non-dominant hand to collect two raw kidney beans from the home cup and transport them to one of the three target cups. The non-dominant hand was used to ensure the task was not overlearned and to avoid potential confounds of a ceiling effect (40). Participants were instructed to move first to the target cup ipsilateral to the non-dominant hand, then to the middle cup, then to the contralateral cup, repeating this pattern four more times. Thus, each trial consisted of 15 reaches. The primary measure of performance was *trial time*, which began when the participant picked up the spoon and ended when they completed all reaching movements and placed the spoon back onto the table; thus, lower trial times indicated better performance. Dropping beans, transporting an incorrect amount, or moving to the wrong target were counted as errors, and the participant could not continue until the error was corrected, therefore errors contributed to longer trial times. Participants were not provided with performance feedback but could explore different movement strategies to optimize performance [i.e., discovery learning (41)]. As noted previously, each training session consisted of 50 trials (i.e., 750 reaches per session), and participants completed three training sessions over 3 days (1/day), totaling 2,250 reaches. This dose of training was selected based on previous feasibility and efficacy studies in other clinical and healthy populations (42, 43).

### Statistical Analysis

JMP Pro 14.0 (SAS Institute Inc., Cary NC) was used for all statistical analyses. To examine whether global cognition was related to the amount of learned motor skill, MoCA scores were included in a multiple linear regression model as a predictor of nine-day follow-up performance (α = 0.05), along with baseline motor performance, age, MDS-UPDRS Motor subsection score, and GDS as covariates. The MDS-UPDRS Motor subsection score and GDS were included to control for severity of PD motor signs and depressive symptoms, respectively. All continuous variables (age, MoCA, baseline motor performance, and 9-day follow-up) were normally distributed, as determined by Shapiro-Wilk tests. Furthermore, a partial correlation matrix indicated minimal collinearity between predictors, with only a moderate correlation between age and MoCA (*r* = −0.21). In addition, quantile range analysis indicated no outliers for any of the continuous variables. Thus, assumptions for linear regression were tested and met.

Baseline motor performance was measured as the first trial of the first motor training session. Similarly, follow-up was measured as a single trial performed 9 days after the last training session. Although we did not have a specific hypothesis regarding the effect of dopamine replacement medication on learning, we also included the variable of group (“on” vs. “off” medication) as a covariate to control for any confounds of dopamine replacement medication status on the primary outcome. We note that baseline motor performance was not different between the medication groups (*p* = 0.28), consistent with results from the primary analysis of this clinical trial (30). We also note that based on results from the primary analysis of this clinical trial [see Table 2 in original publication (30)], we were sufficiently powered to detect significant differences between the two timepoints (Cohen's *d* = 0.58; effect-size *r* = 0.27).

## Results

Individual characteristics are provided in Table 1. Participants demonstrated mild PD symptoms and disease severity (median Hoehn and Yahr stage = 2, not shown in table). Using their non-dominant hand, participants completed the Nine-hole peg test and experimental dexterity task in 25.38 ± 4.31 and 102.81 ± 54.37 s (mean ± SD), respectively. Results for the Nine-hole peg test were consistent with previously reported values in PD (33). In addition, participants were bradykinetic, taking twice as long to complete the dexterity task as healthy older adults from previously reported data (35). MoCA scores ranged between 23 and 30, indicating that some participants were within the normal range of cognition while others fell below [based on (31)].

**Table 1:** Individual participant characteristics (*n* = 23).

**Age (years)**	**Education (years)**	**GDS^**a**^**	**MDS-UPDRS-3^**b**^**	**Gender**	**9HPT (s)^**c**^**	**UE dexterity (s)^**d**^**	**MoCA^**e**^**
66.5	18	10	36	M	30.38	193.66	24
71.2	18	0	34	F	22.5	109.36	30
75.8	12	1	25	F	20.75	42.83	25
68.8	14	3	33	M	22.37	103.11	28
66.5	16	1	32	F	22.85	47.02	27
67.7	17	1	40	M	23.03	70.42	30
50.5	16	1	21	F	19.31	45.86	30
74.4	16	1	39	F	24.19	69.24	25
79.2	18	1	28	M	26.91	103.99	26
71.7	16	0	34	M	28.88	80.89	27
70	18	1	35	M	24.85	107.29	24
62.4	12	1	32	M	25.66	93.27	26
79.6	16	0	22	M	27.31	121.90	25
78.7	16	9	24	F	23.35	63.21	28
77.9	16	0	27	F	25.93	117.21	27
70	16	0	39	F	24.59	100.00	27
80.3	20	10	20	M	27.88	92.90	27
76.2	12	1	37	F	22.52	100.33	24
66.2	20	9	13	F	19.44	61.37	27
73.9	20	0	24	M	28.53	96.05	29
70.8	14	2	27	F	24.44	153.98	28
63.4	20	5	25	F	27.63	94.89	23
72.9	14	3	55	M	39.69	296.02	25

a *GDS, Geriatric Depression Scale*.

b *MDS-UPDRS-3, Movement Disorder Society—Unified Parkinson's Disease Rating Scale Motor Portion (assessed “on” medication)*.

c *9 HPT, Nine Hole Peg Test, tested on the non-dominant hand (prior to motor training); higher scores indicate worse performance, measured in seconds*.

d *UE Dexterity Task, Upper Extremity Dexterity task (non-dominant hand); higher scores indicate worse performance, measured in s*.

e *MoCA, Montreal Cognitive Assessment; lower scores indicate worse performance*.

**Figure 2:**
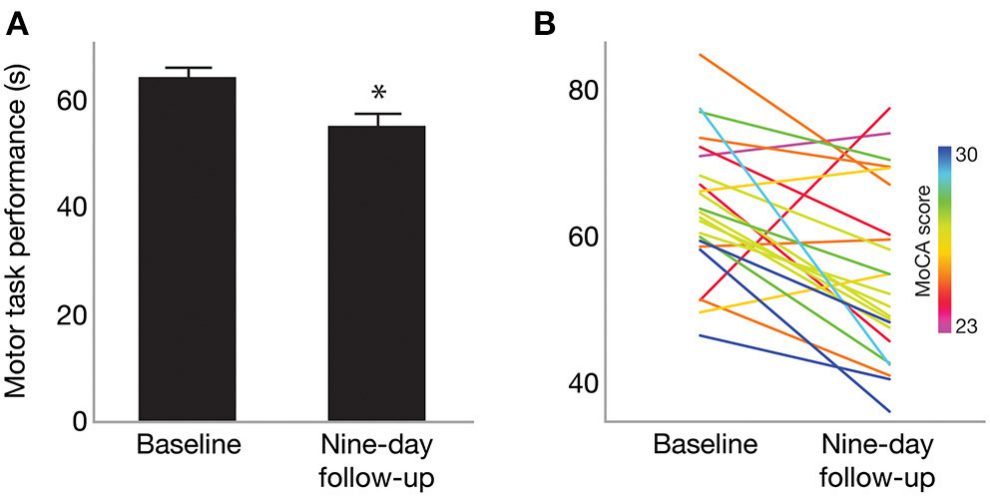
**(A)** Mean motor task performance at baseline and 9-day follow-up. On average, trial time (in seconds) was significantly faster at nine-day follow-up compared to baseline. *Indicates *p* = 0.0016. **(B)** Motor performance for each participant at baseline and 9-day follow-up. Line color indicates each participant's MoCA score, with warmer colors indicating lower MoCA scores and cooler colors indicating higher MoCA scores (range 23–30).

**Figure 3:**
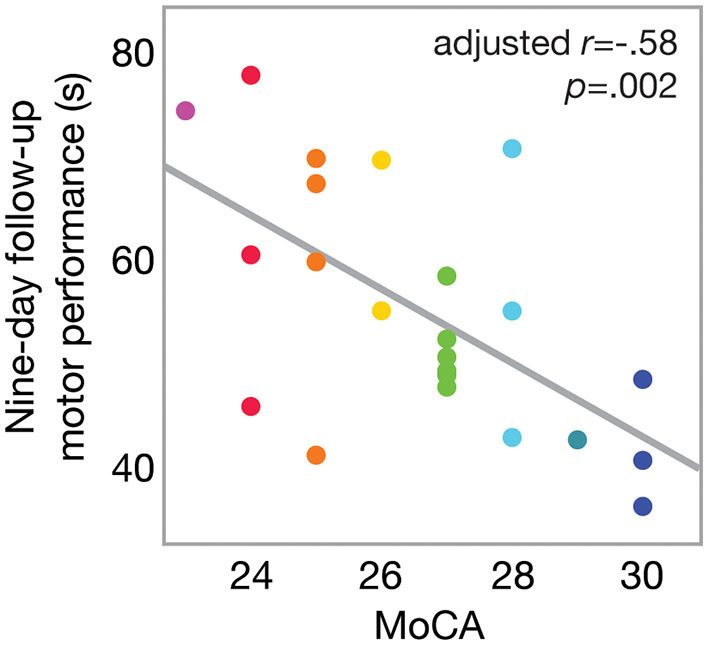
Scatterplot of motor performance at 9-day follow-up as a function of MoCA score. Bivariate linear correlation results are shown in figure. Color gradient corresponds to that in Figure 2.

Overall, nine-day follow-up performance on the motor task was significantly faster (better) than that of baseline [one-sample *t*_(43)_ = −3.13; *p* = 0.0016], as shown in Figure 2A. This indicates an overall effect of motor learning in this sample. Linear regression model results indicated that only MoCA score predicted 9-day follow-up performance (β = −2.76; 95% CI [−5.11, −0.39], *p* = 0.0248), such that higher MoCA scores were associated with faster (better) trial times 9 days post-training. This is further illustrated in Figure 2B, which shows baseline and follow-up data for each participant. Line colors indicate each participant's MoCA score (warmer colors = lower MoCA scores), such that warmer colors tended to cluster at slower trial times for 9-day follow-up, while cooler colors tended to cluster at faster trial times. Participant age (β = −0.18; 95% CI [−0.89, 0.52], *p* = 0.59), severity of PD motor signs (β = 0.26; 95% CI [−0.34, 0.86], *p* = 0.37), GDS (β = 0.71; 95% CI [−0.73, 2.15], *p* = 0.31) and medication status group (β = 2.83; 95% CI [−2.02, 7.68], *p* = 0.23) were not significantly related to follow-up motor performance. Since only MoCA score was a significant predictor of follow-up performance, results from a bivariate regression are provided in Figure 3 to further illustrate the negative relationship between the two variables (color gradient consistent with that of Figure 2B).

## Discussion

The purpose of this secondary analysis was to determine whether cognition was related to upper extremity motor skill learning in individuals with PD. Results indicated that MoCA score predicted follow-up performance of a functional upper extremity motor task 9 days after the last practice session, more so than baseline performance and age (regardless of “on”/“off” medication groups). These findings align with previous work that suggest cognitive testing can be used to predict rehabilitative outcomes (44, 45) and support that global cognition may be a useful tool to predict motor learning in clinical populations (46–48). Although the MoCA was used to evaluate global cognition in this study, there is existing evidence that supports the value of assessing specific cognitive domains as predictors of motor learning (24, 44, 49).

Given the high prevalence of cognitive impairment among people with PD (50, 51), global and specific cognitive measures should be considered to identify which and to what extent various cognitive impairments interfere with learning different skills. In terms of global cognitive measures, the MoCA may be particularly sensitive to screening cognitive deficits associated with PD compared to other global cognitive measures of cognition (i.e., the Mini Mental State Examination) (32). We acknowledge, however, that the MoCA is a rapid cognitive screen that does not thoroughly assess the function of each cognitive domain nor is it validated to measure the function of individual cognitive domains, which may be a limitation to this study. Thus, a more comprehensive battery of cognitive assessments would determine whether specific cognitive domains (or specific cognitive deficits) more closely predict motor skill retention in this population. For example, visuospatial deficits may interfere with upper-extremity learning (23–25, 52, 53), while fluid cognitive skills or executive function may interfere with lower-extremity learning (54). While these previous studies have not focused on motor learning in PD specifically, the effects of particular cognitive deficits may not be PD-specific but instead generalize to a number of older patient populations who may be receiving motor rehabilitation for a number of reasons (e.g., stroke, joint replacement). As such, the effect of cognitive impairment on motor rehabilitation is gaining interest, within and beyond PD (12, 55). Future studies in motor learning should consider a more comprehensive battery of cognitive tests, especially those that evaluate visuospatial and executive abilities, in order to identify evidence-based targets for adjuvant cognitive or non-invasive brain stimulation therapies [e.g. (15, 17, 56–58)] that can be administered prior to or during upper- and lower-extremity motor therapy for people with PD.

In addition to providing empirical evidence as the groundwork for developing effective adjuvant therapies for motor rehabilitation in PD, this study offers clinicians a low-cost, easy-to-implement way to predict how responsive a person with PD might be to motor therapy. It is well-established that responsiveness to motor rehabilitation is often highly variable between PD patients [see 95% CIs in Robinson et al. (9)]. As such, the findings from the current study suggest that the MoCA may be a quick (~5–10 min) and simple way to predict how responsive a patient might be to upper-extremity training. This would inform therapists in how to streamline and tailor their treatments, and better allocate their time to activities that they know their patients will benefit from. Predictors of therapeutic responsiveness are already being explored outside PD using neuroimaging (59–61), neurophysiology (61–63), or genotyping (64–66), but these investigational methods are time- and cost-intensive, making them unfeasible for an allied health setting and out-of-pocket therapy.

In the published clinical trial (30), there was a modest effect of medication status during training (i.e., “on”/“off” medication while practicing the task) between baseline and 48-h follow-up task performance, such that the “on” medication group performed significantly better at this short-term retention period than did the “off” medication group. These results were interpreted to indicate that being “on” dopamine replacement medications may facilitate short term retention of motor skill. However, this secondary analysis indicates that the group difference was no longer present by the ninth day of retention, likely due to the modest effect of medication status on training previously observed. Instead, global cognition (which was not originally considered in the parent clinical trial) was a significant predictor of motor task performance well after training had been completed (9 days later), regardless of whether training had occurred “on” or “off” dopamine replacement medication. Indeed, dopamine replacement may be insufficient to offset the breadth of cognitive deficits associated with PD (67), and the short duration in which participants in the “off” group were withdrawn from their medication for training (relative to the 9-day duration of retention) may explain the lack of effect of group in this secondary analysis.

There are several limitations to this study. First, there was a limited range of MoCA scores in this sample, and more participants were in the normal range than below. Even though the MoCA is not a diagnostic tool (and is instead a cognitive screening tool), scores suggest that the majority of participants were cognitively intact, and more extensive neuropsychological testing would be necessary to determine whether participants with scores below the “normal” cut-off were in fact cognitively-impaired. This does not, however, take away from the findings and implications of this study, whereby rehabilitation-focused clinicians could still use the MoCA as a quick screening tool to better predict how patients might respond to upper-extremity skill training. Future studies will investigate a larger cohort of more cognitively-impaired individuals [such as people with PD who experience “freezing” (68–71)] to further test the generalizability of these findings. Second, a limitation of this study is that the participants in the “off” medication group resumed their regularly prescribed dopamine replacement therapy after training each day and throughout the 9-day retention period; thus, we are unable to discern potential effects of medication adherence or withdrawal on motor skill consolidation and retention. It is well-established that consolidation and retention are critical periods for motor learning, as well as acquisition (72). Third, we acknowledge that this study was not designed to directly test if global cognition would be predictive of clinical rehabilitation motor outcomes in individuals with PD, since it only evaluated the amount of skill retained over a period of 9 days. Performance of the functional upper extremity task used in this study has, however, been associated with subjective and objective measures of daily functioning in individuals diagnosed with Mild Cognitive Impairment (39), suggesting that the benefits of training may generalize to activities of daily living.

## Conclusions

Our study supports the premise that cognitive impairments interfere with motor skill learning in PD, and provides the proof-of-principle that (1) cognitive screening may be a viable solution for personalizing motor rehabilitation for people with PD and (2) cognitive therapy and/or brain stimulation prior to, or concurrent with, motor training could enhance functional outcomes. Future mechanistic work should systematically test which specific cognitive domains are most relevant for different types of motor learning in PD to help inform targeted adjuvant cognitive or neurostimulation therapies that can enhance motor rehabilitation. For example, fluid cognition training may enhance gait adaptation, or non-invasive stimulation of parietal cortex could enhance functional upper-extremity training via visuospatial processes.

## Data Availability Statement

Meta-data are available upon reasonable request. Requests to access these datasets should be directed to lee.dibble@hsc.utah.edu.

## Ethics Statement

The studies involving human participants were reviewed and approved by University of Utah Institutional Review Board. The patients/participants provided their written informed consent to participate in this study.

## Author Contributions

LD and SP organized the database. JLV and SS performed the statistical analysis. JLV wrote the first draft with assistance from CL-L. All authors contributed to conception and design of the study and contributed to manuscript revision, read, and approved the submitted version.

## Funding

This work was supported in part by the National Institute on Aging at the National Institutes of Health (K01 AG047926 to SS and F31 AG062057 to JLV); University of Utah Office of Research (2015-16 grant), USA; the American Parkinson's Disease Association (2015-16 postdoctoral fellowship grant).

## Conflict of Interest

The authors declare that the research was conducted in the absence of any commercial or financial relationships that could be construed as a potential conflict of interest.

## Publisher's Note

All claims expressed in this article are solely those of the authors and do not necessarily represent those of their affiliated organizations, or those of the publisher, the editors and the reviewers. Any product that may be evaluated in this article, or claim that may be made by its manufacturer, is not guaranteed or endorsed by the publisher.

## References

[1] Nowak DA Hermsdörfer J. Objective evaluation of manual performance deficits in neurological movement disorders. Brain Res Rev. (2006) 51:108–24. 10.1016/j.brainresrev.2005.10.003 16356552

[2] Ingvarsson PE Gordon AM Forssberg H. Coordination of manipulative forces in Parkinson's disease. Exp Neurol. (1997) 145:489–501. 10.1006/exnr.1997.6480 9217085

[3] Raggi A Leonardi M Ajovalasit D Carella F Soliveri P Albanese A. Disability and profiles of functioning of patients with Parkinson's disease described with ICF classification. Int J Rehabil Res. (2011) 34:141–50. 10.1097/MRR.0b013e328344ae09 21427589

[4] Vanbellingen T Nyffeler T Nigg J Janssens J Hoppe J Nef T. Home based training for dexterity in Parkinson's disease: a randomized controlled trial. Parkinsonism Relat Disord. (2017) 41:92–8. 10.1016/j.parkreldis.2017.05.021 28578819

[5] Nackaerts E Vervoort G Heremans E Smits-Engelsman BCM Swinnen SP Nieuwboer A. Relearning of writing skills in Parkinson's disease: a literature review on influential factors and optimal strategies. Neurosci Biobehav Rev. (2013) 37:349–57. 10.1016/j.neubiorev.2013.01.015 23333265

[6] Nieuwboer A Rochester L Müncks L Swinnen SP. Motor learning in Parkinson's disease: limitations and potential for rehabilitation. Parkinsonism Relat Disord. (2009) 15:S53–8. 10.1016/S1353-8020(09)70781-3 20083008

[7] Felix K Gain K Paiva E Whitney K Jenkins ME Spaulding SJ. Upper extremity motor learning among individuals with Parkinson's disease: a meta-analysis evaluating movement time in simple tasks. Parkinsons Dis. (2012) 2012:589152. 10.1155/2012/589152 22191071 PMC3236460

[8] Mak MK Wong-Yu IS Shen X Chung CL. Long-term effects of exercise and physical therapy in people with Parkinson disease. Nat Rev Neurol. (2017) 13:689–703. 10.1038/nrneurol.2017.128 29027544

[9] Robinson AG Dennett AM Snowdon DA. Treadmill training may be an effective form of task-specific training for improving mobility in people with Parkinson's disease and multiple sclerosis: a systematic review and meta-analysis. Physiotherapy. (2019) 105:174–86. 10.1016/j.physio.2018.11.007 30876717

[10] Salmanpour MR Shamsaei M Saberi A Klyuzhin IS Tang J Sossi V. Machine learning methods for optimal prediction of motor outcome in Parkinson's disease. Phys Med. (2020) 69:233–40. 10.1016/j.ejmp.2019.12.022 31918375

[11] Leung KH Salmanpour MR Saberi A Klyuzhin IS Sossi V Jha AK. Using deep-learning to predict outcome of patients with Parkinson's disease. In: *IEEE Nuclear Science Symposium and Medical Imaging Conference Proceedings (NSS/MIC)*. (2018). p. 1–4. 10.1109/NSSMIC.2018.8824432

[12] Van Gilder JL Hooyman A Peterson DS Schaefer SY. Post-stroke cognitive impairments and responsiveness to motor rehabilitation: a review. Curr Phys Med Rehabil Rep. (2020) 8:461–8. 10.1007/s40141-020-00283-3 33767922 PMC7987128

[13] Schaefer SY Sullivan JM Peterson DS Fauth EB. Cognitive function at admission predicts amount of gait speed change in geriatric physical rehabilitation. Ann Phys Rehabil Med. (2020) 63:359–61. 10.1016/j.rehab.2019.08.004 31520785 PMC7367220

[14] Friedman PJ Baskett JJ Richmond DE. Cognitive impairment and its relationship to gait rehabilitation in the elderly. N Z Med J. (1989) 102:603–6. 2594278

[15] Jung SH Hasegawa N Mancini M King LA Carlson-Kuhta P Smulders K. Effects of the agility boot camp with cognitive challenge (ABC-C) exercise program for Parkinson's disease. NPJ Parkinsons Dis. (2020) 6:31. 10.1038/s41531-020-00132-z 33298934 PMC7608677

[16] Avenali M Picascia M Tassorelli C Sinforiani E Bernini S. Evaluation of the efficacy of physical therapy on cognitive decline at 6-month follow-up in Parkinson disease patients with mild cognitive impairment: a randomized controlled trial. Aging Clin Exp Res. (2021). 10.1007/s40520-021-01865-4. [Epub ahead of print]. 33978924

[17] Bernini S Alloni A Panzarasa S Picascia M Quaglini S Tassorelli C. A computer-based cognitive training in mild cognitive impairment in Parkinson's disease. NeuroRehabilitation. (2019) 44:555–67. 10.3233/NRE-192714 31256092

[18] Bo J Seidler RD. Visuospatial working memory capacity predicts the organization of acquired explicit motor sequences. J Neurophysiol. (2009) 101:3116–25. 10.1152/jn.00006.2009 19357338 PMC2694099

[19] Langan J Seidler RD. Age differences in spatial working memory contributions to visuomotor adaptation and transfer. Behav Brain Res. (2011) 225:160–8. 10.1016/j.bbr.2011.07.014 21784106 PMC3170505

[20] Jeunet C Jahanpour E Lotte F. Why standard brain-computer interface (BCI) training protocols should be changed: an experimental study. J Neural Eng. (2016) 13:36024. 10.1088/1741-2560/13/3/036024 27172246

[21] Jeunet C Kaoua B Subramanian S Hachet M Lotte F. Predicting mental imagery-based BCI performance from personality, cognitive profile and neurophysiological patterns. PLoS ONE. (2015) 10:e0143962. 10.1371/journal.pone.0143962 26625261 PMC4666487

[22] Bo J Borza V Seidler RD. Age-related declines in visuospatial working memory correlate with deficits in explicit motor sequence learning. J Neurophysiol. (2009) 102:2744–54. 10.1152/jn.00393.2009 19726728 PMC2777814

[23] Van Gilder JL Hengge CR Duff K Schaefer SY. Visuospatial function predicts one-week motor skill retention in cognitively intact older adults. Neurosci Lett. (2018) 664:139–43. 10.1016/j.neulet.2017.11.032 29154858 PMC5817029

[24] Van Gilder JL Walter CS Hengge CR Schaefer SY. Exploring the relationship between visuospatial function and age-related deficits in motor skill transfer. Aging Clin Exp Res. (2020) 32:1451–8. 10.1007/s40520-019-01345-w 31520336 PMC7067619

[25] Van Gilder JL Lohse KR Duff K Wang P Schaefer SY. Evidence for associations between Rey-Osterrieth Complex Figure test and motor skill learning in older adults. Acta Psychol (Amst). (2021) 214:103261. 10.1016/j.actpsy.2021.103261 33524606 PMC7920933

[26] Chan JSY Wu Q Liang D Yan JH. Visuospatial working memory training facilitates visually-aided explicit sequence learning. Acta Psychol. (2015) 161:145–53. 10.1016/j.actpsy.2015.09.008 26398484

[27] Mata IF Leverenz JB Weintraub D Trojanowski JQ Chen-Plotkin A Van Deerlin VM. GBA Variants are associated with a distinct pattern of cognitive deficits in Parkinson's disease. Mov Disord. (2016) 31:95–102. 10.1002/mds.26359 26296077 PMC4724255

[28] Cummings JL. The dementias of Parkinson's disease: prevalence, characteristics, neurobiology, and comparison with dementia of the Alzheimer type. Eur Neurol. (1988) 28:15–23. 3288478

[29] Sahakian BJ Morris RG Evenden JL Heald A Levy R Philpot M. A comparative study of visuospatial memory and learning in Alzheimer-type dementia and Parkinson's disease. Brain. (1988) 111:695–718. 10.1093/brain/111.3.695 3382917

[30] Paul SS Dibble LE Olivier GN Walter C Duff K Schaefer SY. Dopamine replacement improves motor learning of an upper extremity task in people with Parkinson disease. Behav Brain Res. (2020) 377:112213. 10.1016/j.bbr.2019.112213 31526767 PMC7398159

[31] Nasreddine ZS Phillips NA Bédirian V Charbonneau S Whitehead V Collin I. The Montreal Cognitive Assessment, MoCA: a brief screening tool for mild cognitive impairment. J Am Geriatr Soc. (2005) 53:695–9. 10.1111/j.1532-5415.2005.53221.x 15817019

[32] Hoops S Nazem S Siderowf AD Duda JE Xie SX Stern MB. Validity of the MoCA and MMSE in the detection of MCI and dementia in Parkinson disease. Neurology. (2009) 73:1738–45. 10.1212/WNL.0b013e3181c34b47 19933974 PMC2788810

[33] Earhart GM Cavanaugh JT Ellis T Ford MP Foreman KB Dibble L. The 9-hole PEG test of upper extremity function: average values, test-retest reliability, and factors contributing to performance in people with Parkinson disease. J Neurol Phys Ther. (2011) 35:157–63. 10.1097/NPT.0b013e318235da08 22020457

[34] Schaefer SY. Preserved motor asymmetry in late adulthood: is measuring chronological age enough? Neuroscience. (2015) 294:51–9. 10.1016/j.neuroscience.2015.03.013 25772792

[35] Walter CS Hengge CR Lindauer BE Schaefer SY. Declines in motor transfer following upper extremity task-specific training in older adults. Exp Gerontol. (2019) 116:14–9. 10.1016/j.exger.2018.12.012 30562555 PMC6339591

[36] Goetz CG Tilley BC Shaftman SR Stebbins GT Fahn S Martinez-Martin P. Movement Disorder Society-sponsored revision of the Unified Parkinson's Disease Rating Scale (MDS-UPDRS): scale presentation and clinimetric testing results. Mov Disord. (2008) 23:2129–70. 10.1002/mds.22340 19025984

[37] Yesavage JA Sheikh JI. 9/Geriatric Depression Scale (GDS). Clin Gerontol. (1986) 5:165–73. 10.1300/J018v05n01_0922588766

[38] Katz S Downs TD Cash HR Grotz RC. Progress in development of the Index of ADL1. Gerontologist. (1970) 10:20–30. 10.1093/geront/10.1_Part_1.20 5420677

[39] Schaefer SY Hooyman A Duff K. Using a timed motor task to predict one-year functional decline in amnestic mild cognitive impairment. J Alzheimers Dis. (2020) 77:53–8. 10.3233/JAD-200518 32651327 PMC7484390

[40] Suchy Y Kraybill ML Franchow E. Practice effect and beyond: reaction to novelty as an independent predictor of cognitive decline among older adults. J Int Neuropsychol Soc. (2011) 17:101–11. 10.1017/S135561771000130X 21073771

[41] Orrell AJ Eves FF Masters RSW. Implicit motor learning of a balancing task. Gait Posture. (2006) 23:9–16. 10.1016/j.gaitpost.2004.11.010 16311189

[42] Schaefer SY Dibble LE Duff K. Efficacy and feasibility of functional upper extremity task-specific training for older adults with and without cognitive impairment. Neurorehabil Neural Repair. (2015) 29:636–44. 10.1177/1545968314558604 25416739

[43] Schaefer SY Patterson CB Lang CE. Transfer of training between distinct motor tasks after stroke. Neurorehabil Neural Repair. (2013) 27:602–12. 10.1177/1545968313481279 23549521 PMC3769167

[44] Toglia J Fitzgerald KA O'Dell MW Mastrogiovanni AR Lin CD. The mini-mental state examination and montreal cognitive assessment in persons with mild subacute stroke: relationship to functional outcome. Arch Phys Med Rehabil. (2011) 92:792–8. 10.1016/j.apmr.2010.12.034 21530727

[45] Saverino A Waller D Rantell K Parry R Moriarty A Playford ED. The role of cognitive factors in predicting balance and fall risk in a neuro-rehabilitation setting. PLoS ONE. (2016) 11:e0153469. 10.1371/journal.pone.0153469 27115880 PMC4846032

[46] Zietemann V Georgakis MK Dondaine T Müller C Mendyk A-M Kopczak A. Early MoCA predicts long-term cognitive and functional outcome and mortality after stroke. Neurology. (2018) 91:e1838 LP-e1850. 10.1212/WNL.0000000000006506 30333158

[47] Lim K-B Kim J Lee H-J Yoo J You E-C Kang J. Correlation between montreal cognitive assessment and functional outcome in subacute stroke patients with cognitive dysfunction. Ann Rehabil Med. (2018) 42:26–34. 10.5535/arm.2018.42.1.26 29560321 PMC5852226

[48] Abzhandadze T Rafsten L Lundgren Nilsson Å Palstam A Sunnerhagen KS. Very early MoCA can predict functional dependence at 3 months after stroke: a longitudinal, cohort study. Front Neurol. (2019) 10:1051. 10.3389/fneur.2019.01051 31681142 PMC6798188

[49] Wang P Infurna FJ Schaefer SY. Predicting motor skill learning in older adults using visuospatial performance. J Mot Learn Dev. (2019) 8:38–51. 10.1123/jmld.2018-0017 34109252 PMC8186454

[50] Hely MA Reid WGJ Adena MA Halliday GM Morris JGL. The Sydney multicenter study of Parkinson's disease: the inevitability of dementia at 20 years. Mov Disord. (2008) 23:837–44. 10.1002/mds.21956 18307261

[51] Aarsland D Brønnick K Larsen JP Tysnes OB Alves G. Cognitive impairment in incident, untreated Parkinson disease: the Norwegian ParkWest study. Neurology. (2009) 72:1121–6. 10.1212/01.wnl.0000338632.00552.cb 19020293

[52] Van Gilder JL Hooyman A Bosch PR Schaefer SY. Generalizing the predictive relationship between 1-month motor skill retention and Rey-Osterrieth Delayed Recall scores from nondemented older adults to individuals with chronic stroke: a short report. J Neuroeng Rehabil. (2021) 18:94. 10.1186/s12984-021-00886-4 34082761 PMC8173502

[53] Mullick AA Subramanian SK Levin MF. Emerging evidence of the association between cognitive deficits and arm motor recovery after stroke: a meta-analysis. Restor Neurol Neurosci. (2015) 33:389–403. 10.3233/RNN-150510 26410581 PMC4923759

[54] French MA Cohen ML Pohlig RT Reisman DS. Fluid cognitive abilities are important for learning and retention of a new, explicitly learned walking pattern in individuals after stroke. Neurorehabil Neural Repair. (2021) 35:419–30. 10.1177/15459683211001025 33754890 PMC8122051

[55] McDonald MW Black SE Copland DA Corbett D Dijkhuizen RM Farr TD. Cognition in stroke rehabilitation and recovery research: consensus-based core recommendations from the second stroke recovery and rehabilitation roundtable. Neurorehabil Neural Repair. (2019) 33:943–50. 10.1177/1545968319886444 31660787

[56] Begemann MJ Brand BA Curčić-Blake B Aleman A Sommer IE. Efficacy of non-invasive brain stimulation on cognitive functioning in brain disorders: a meta-analysis. Psychol Med. (2020) 50:2465–86. 10.1017/S0033291720003670 33070785 PMC7737055

[57] King LA Mancini M Smulders K Harker G Lapidus JA Ramsey K. Cognitively challenging agility boot camp program for freezing of gait in Parkinson Disease. Neurorehabil Neural Repair. (2020) 34:417–27. 10.1177/1545968320909331 32249668 PMC7217755

[58] Jiang Y Guo Z McClure MA He L Mu Q. Effect of rTMS on Parkinson's cognitive function: a systematic review and meta-analysis. BMC Neurol. (2020) 20:377. 10.1186/s12883-020-01953-4 33076870 PMC7574251

[59] Cirstea CM Lee P Craciunas SC Choi I-Y Burris JE Nudo RJ. Pre-therapy neural state of bilateral motor and premotor cortices predicts therapy gain after subcortical stroke: a pilot study. Am J Phys Med Rehabil. (2018) 97:23–33. 10.1097/PHM.0000000000000791 28737516 PMC5736430

[60] Tozlu C Edwards D Boes A Labar D Tsagaris KZ Silverstein J. Machine learning methods predict individual upper-limb motor impairment following therapy in chronic stroke. Neurorehabil Neural Repair. (2020) 34:428–39. 10.1177/1545968320909796 32193984 PMC7217740

[61] Burke Quinlan E Dodakian L See J McKenzie A Le V Wojnowicz M. Neural function, injury, and stroke subtype predict treatment gains after stroke. Ann Neurol. (2015) 77:132–45. 10.1002/ana.24309 25382315 PMC4293339

[62] Stinear CM Byblow WD Ackerley SJ Smith M-C Borges VM Barber PA. Proportional motor recovery after stroke: implications for trial design. Stroke. (2017) 48:795–8. 10.1161/STROKEAHA.116.016020 28143920

[63] Smith M-C Byblow WD Barber PA Stinear CM. Proportional recovery from lower limb motor impairment after stroke. Stroke. (2017) 48:1400–3. 10.1161/STROKEAHA.116.016478 28341754

[64] Qin L Jing D Parauda S Carmel J Ratan RR Lee FS. An adaptive role for BDNF Val66Met polymorphism in motor recovery in chronic stroke. J Neurosci. (2014) 34:2493–502. 10.1523/JNEUROSCI.4140-13.2014 24523540 PMC3921423

[65] Pham M Van Miyaguchi S Watanabe H Saito K Otsuru N Onishi H. Effect of repetitive passive movement before motor skill training on corticospinal excitability and motor learning depend on BDNF polymorphisms. Front Hum Neurosci. (2021) 15:621358. 10.3389/fnhum.2021.621358 33633556 PMC7901944

[66] Kim E-J Park C-H Chang WH Lee A Kim ST Shin Y-I. The brain-derived neurotrophic factor Val66Met polymorphism and degeneration of the corticospinal tract after stroke: a diffusion tensor imaging study. Eur J Neurol. (2016) 23:76–84. 10.1111/ene.12791 26228236

[67] Kulisevsky J García-Sánchez C Berthier ML Barbanoj M Pascual-Sedano B Gironell A. Chronic effects of dopaminergic replacement on cognitive function in Parkinson's disease: a two-year follow-up study of previously untreated patients. Mov Disord. (2000) 15:613–26. 10.1002/1531-8257(200007)15:4<613::aid-mds1005>3.0.co;2-f 10928571

[68] Ginis P Heremans E Ferrari A Bekkers EMJ Canning CG Nieuwboer A. External input for gait in people with Parkinson's disease with and without freezing of gait: one size does not fit all. J Neurol. (2017) 264:1488–96. 10.1007/s00415-017-8552-6 28653213

[69] Hall JM Shine JM Walton CC Gilat M Kamsma YPT Naismith SL. Early phenotypic differences between Parkinson's disease patients with and without freezing of gait. Parkinsonism Relat Disord. (2014) 20:604–7. 10.1016/j.parkreldis.2014.02.028 24679901

[70] Peterson DS Fling BW Mancini M Cohen RG Nutt JG Horak FB. Dual-task interference and brain structural connectivity in people with Parkinson's disease who freeze. J Neurol Neurosurg Psychiatry. (2015) 86:786–92. 10.1136/jnnp-2014-308840 25224677 PMC4363035

[71] Cancela JM Nascimento CM Varela S Seijo-Martinez M Lorenzo-Lopez L Millan-Calenti JC. Influence of cognitive impairment on the freezing of gait in non-demented people with Parkinson's disease. Rev Neurol. (2018) 66:289–96. 10.33588/rn.6609.2017289 29696615

[72] Kantak SS Winstein CJ. Learning-performance distinction and memory processes for motor skills: a focused review and perspective. Behav Brain Res. (2012) 228:219–31. 10.1016/j.bbr.2011.11.028 22142953

